# Double bookkeeping and schizophrenia spectrum: divided unified phenomenal consciousness

**DOI:** 10.1007/s00406-020-01185-0

**Published:** 2020-09-08

**Authors:** Josef Parnas, Annick Urfer-Parnas, Helene Stephensen

**Affiliations:** 1 Center for Subjectivity Research, University of Copenhagen, 2300 Copenhagen, Denmark; 2 Mental Health Centre Glostrup, University Hospital of Copenhagen, 2605 Brøndby, Denmark; 3 Faculty of Health and Medical Sciences, University of Copenhagen, 2200 Copenhagen, Denmark; 4 Mental Health Centre Amager, University Hospital of Copenhagen, 1610 Copenhagen, Denmark

**Keywords:** Schizophrenia spectrum, Double bookkeeping, Auto-affection, Self-disorders, Consciousness

## Abstract

Eugen Bleuler, the founder of the concept of schizophrenia, pointed out that psychotic patients were able to live in two disjoint worlds (namely, the social, intersubjective world and the delusional world). He termed this phenomenon “double bookkeeping,” but did not provide any conceptual elaboration of this phenomenon or its possible mechanisms. Double bookkeeping has been neglected in mainstream psychiatry, but it has been addressed in recent theoretical work, however mainly concerned with the issue of delusion. In this article, we present clinical material that supports the view that double bookkeeping manifests itself across various psychotic phenomena and its antecedent may be observed in premorbid (pre-onset) phases as well as in the schizotypal disorder. We try to conceptualize double bookkeeping to concretize an often atmospheric perception of paradoxicality in the encounter with the patient. A phenomenological analysis of double bookkeeping suggests an instability in the affective (“auto-affection”) articulation of selfhood. We point to four main implications of our presentation: (1) diagnostic, (2) epistemological, (3) therapeutic and (4) pathogenetic research.

## Introduction

Clinical observations of the phenomenon of double bookkeeping, although not conceptualized with this term, can already be found in the works of Philippe Pinel [1] and Jean-Étienne Esquirol [2]. The notion of double bookkeeping was coined by Eugen Bleuler in his monograph on schizophrenia [3] and his subsequent textbook of psychiatry [4] referring to the patients’ ability to separate their delusional world from the everyday socially shared world. According to Bleuler, this reflects a co-existence of two disjoint ways of orienting oneself to reality. This is well illustrated in the following quote from Bleuler.

Kings and Emperors, Popes, and Redeemers engage, for the most part, in quite banal work.[…]. None of our generals has ever attempted to act in accordance with his imaginary rank and station [3, p. 129].

As an example, from our own clinical work, we can mention a hospitalized patient, who claims that the nurses are trying to poison him, but he nonetheless gladly consumes the food that he is served by the very same personnel. As in the example from Bleuler, the patient does not act according to the content of his delusional experience.

Bleuler introduces the concept of “double entry book-keeping” in the beginning of his book in the section of “intact simple functions” where he argues against a view of schizophrenia as a deficit of delimited cognitive capacities [3]. He observed that even when patients are absorbed in their psychotic experiences, nearly impossible to interact with, they are nonetheless quite acutely aware of what is happening in the shared-social world. One can find examples of double bookkeeping throughout the entire text of his monograph. However, he does not offer any clear definition of double bookkeeping, its potential phenomenological unity or psychological mechanism.

Even though the phenomenon is probably very well known to most experienced clinicians (though not in an explicit or conceptual way), it is completely neglected in contemporary mainstream psychiatry.

However, in the last 10 years we have witnessed emerging interest in the phenomenon of double bookkeeping [5–8]. These contributions deal mainly with theoretical issues concerning delusion and are primarily based on autobiographical narratives of patients with schizophrenia (especially on Schreber’s memoires [9]). In contrast to mainstream psychiatry, the basic idea in these latter studies is that the patient’s experience of the world must not simply be mistaken, but somehow altered or transformed in a global way.

In this paper, we will address double bookkeeping as a situation in which the patient simultaneously lives in two different levels of reality. One reality is our shared, social, mundane (ontic) world with its implicit understanding of the laws of nature, mind-independence of the so-called “external world” and the principle of non-contradiction. The other reality involves a private framework that violates spatio-temporal and non-contradiction constrains of the intersubjective world. It is crucial to emphasize already at this point that the latter form of reality should not be considered as some kind of fiction, fantasy or imagination on the part of the patient. Rather, it possesses for her a significance of reality that is even more true and profound than the socially accepted reality, touching upon ontological structures.^1^

Double bookkeeping is not simply a reflection of harboring conflicting attitudes. Most people do in fact have inconsistent beliefs about different matters, but those beliefs are concordant with normatively acceptable rules of reasoning (e.g., one can be an ardent advocate of equal redistribution of wealth in society while at the same time adhere to the radical tenets of unrestricted capitalism)*.*

Our paper is inspired by a clinical perspective, formed during long-standing empirical and theoretical research and clinical work with patients suffering from the schizophrenia spectrum disorders.

Our main proposal is that double bookkeeping manifests itself before the onset of overt psychosis, in the schizotypal disorders and across a manifold of characteristic psychotic symptoms. We claim that this phenomenon is associated with a certain structural alteration of phenomenal consciousness.

We will therefore start with the clinical exposition of the manifold manifestations of double bookkeeping across different psychopathological phenomena. In the discussion section, we will attempt to identify a shared psychopathological pattern that is indicative of double bookkeeping. Finally, while situating our analysis in an historical framework, we will present a phenomenological analysis of double bookkeeping, as being linked to a specific disorder of selfhood that functions as a precondition of the formation of schizophrenia-specific psychopathology. A corollary of this analysis will entail a critique of the views of schizophrenia as a dissociative disorder akin to multiple personality disorder.

At the end of the paper, we will point to important implications of a phenomenological grasp of double bookkeeping for treatment and empirical research in schizophrenia spectrum disorders.

## Clinical manifestations of double bookkeeping

Double bookkeeping appears across manifold phenomena of schizophrenia such as delusions, hallucinations, behaviors, existential orientation, and the nature of the insight into illness.

It is important to emphasize that in the descriptions of double bookkeeping we do not merely encounter a psychotic patient, who by necessity is forced to live in our common social world. Double bookkeeping is not a contingent feature of schizophrenia in a manner similar to the content of delusional beliefs, e.g., the patient feels persecuted by the CIA, rather than by the KGB. Moreover, it is important to note that this phenomenon of double bookkeeping is most clearly observed and informative in non-acute, stable patients or in patients in the initial stages of their illness. In the acute psychosis with flamboyant symptomatology, the patients tend to conflate their psychotic world with the shared world and may enact their psychotic experiences in the immediate environment.

In the following, we will present selected clinical manifestations of double bookkeeping in different domains of psychopathology, which we have divided into delusions, hallucinations, insight into illness, and *Anderssein* (“being different”). Such separate presentation is useful for didactic reason but we need to remember that the apparently distinct domains strongly overlap and mutually entail each other.

### Delusions

To make clear how double bookkeeping appears in relation to delusions it is necessary to clarify the nature of delusions, which are characteristic of schizophrenia. In continental psychopathology, there is an agreement that a specific nature of delusion in schizophrenia is quite emblematic for this illness [10–15]. It is crucial to emphasize that this approach differs from the mainstream psychiatric definition of delusions as some sort of erroneous belief about the “external world” [16], or as already Karl Jaspers stated it:

To say simply that a delusion is a mistaken idea […] gives only a superficial and incorrect answer to the problem […]. Delusion proper, however, implies a transformation in our total awareness of reality [17, p. 93–95].

Jaspers called the delusions, that are characteristic for schizophrenia, “primary” or “true” and distinguished them from “secondary” delusions (i.e., delusions-like ideas) [17]. Primary delusions were not accessible to a common sense understanding and were therefore not reducible to other psychological phenomena. In contrast, secondary delusions were diagnostically non-specific (occurring both in schizophrenia and in other psychoses) and could be understood as arising from other factors, e.g., delusional guilt in melancholia or systematized delusions emerging upon a paranoid personality organization.

In the following, we will address the nature of the epistemic and existential status of two distinctive features of delusions in schizophrenia: (1) mode of emergence and conviction and, (2) their typical content.

1) Primary delusions in schizophrenia originate in an affective, pathic experience, i.e., the delusional meaning is revealed to the patient in an imposing manner rather than being grasped through cognitive efforts [18, 19]. During the formation of delusions, there is frequently an increase of basic affective tension followed by a crystallization of delusional conviction and insipient meaning [20]. This crystallization is not a product of a step by step inferential reasoning or reflection, but possesses a character of immediacy and revelation.

Case 1: One of our patients with schizophrenia, a 22-year old male, reported of the onset of his illness in the following way: One evening he met some old friends in an amusement park in Copenhagen and during this encounter, he was overwhelmed by a global feeling of intense happiness. On the way home, he suddenly got a thought that he was perhaps a savior, destined to bring peace in the world. This idea formed the basis of subsequent delusional elaborations.

Such revelation is originally an affective, pathic experience with only vague meaning, but carries with itself an absolute affective conviction that precedes the concretization of the delusional content. As the German psychiatrist Hemmo Müller-Suur writes, the delusional conviction in schizophrenia emerges immediately [21]. The consciousness of conviction (*Gewissheitsbewusstsein*) precedes its infusion with a specific content of what one is convinced about. In other words, the patient is convinced that something is happening, but he is not aware of what is exactly happening. This is the essence of the delusional mood [20, 22]. The experience of revelation becomes gradually transformed into a standard subject-object structure. In contrast to schizophrenia continues Müller-Suur, in paranoia, delusional disorder, the conviction is a product of a laborious step by step inferential cognitive process.

2) The content of typical delusions in schizophrenia is frequently colored by metaphysical, eschatological, or charismatic themes [11, 23]. The latter refers to issues concerning the meaning and purpose of human life, where patients may feel to have a central position, to be chosen for a special mission where the meaning of their life reveals itself to them (“charisma” means divine gift). The former refers to issues concerning respectively the essence of Being or existence (i.e., the schizophrenia cosmology is often of a magical character, consisting of a struggle between good and evil forces, or is penetrated by energies, rays, waves and so forth) and ultimate issues such as universal peace or the end of the world. Along the same line, Sass proposed that delusions in schizophrenia rather than being concerned with the mundane (ontic) issues focus on the very (ontological) horizons of human existence [24]. He emphasizes that the patient lives in a double reality with his delusional conviction forming a part of the reality with a “subjectivized” quality that is unconnected to the intersubjective world [5].

In a similar vein, the French psychiatrist Arthur Tatossian and the German psychiatrist Manfred Spitzer emphasize the “egological” nature of delusions in schizophrenia, i.e., comparable to the certitude of having a thought or feeling pain. Accordingly, delusions are reports of private, immanent experience affecting the self, rather than being statements about affairs in the public world [10, 16, 18, 25, 26].^2^

Thus, we have touched upon the epistemic and existential nature of delusions, since the content of the delusions typically reflect this very nature, or as Müller-Suur put it, form and content are dialectically interrelated [29].

In connection to double bookkeeping it is striking that even though primary delusions are in no way corrigible, because of the delusional conviction described above, they are usually never enacted. Jaspers described it in the following way:

Reality does not carry always the same meaning as that of normal reality. With these patients, persecution does not always appear quite like the experience of people who are in fact being persecuted; nor does their jealousy seem like of some justifiably jealous persons […]. Hence, the attitude of the patient to the content of his delusion is peculiarly inconsequent at times [17, p. 105].

These “inconsequential attitudes” concerning respectively a “delusional” and “empirical” reality are illustrated in the following vignette:

Case 2: One of our patients from an open ward claimed that the hospital was surrounded by CIA agents only waiting to kill him. Nonetheless, he went to buy an ice cream apparently undisturbed in a kiosk outside the hospital.

In contrast to schizophrenia, delusions in delusional disorder (paranoia) possess a clearly mundane, “empirical” character and involves mediation by reflective processes. The delusion in this case of paranoia is integrated in our shared social world and typically does not violate natural laws although the latter may be modified to support delusional content.

Case 3: A 50-year-old woman living in own house complained about the quality of the running water; she had an impression that the water was somehow toxic and caused her skin problems and a general malaise. She frequently visited own GP and complained about it. There were several technical inspections from the municipality which did not find anything wrong with the quality of the water. The patient was absorbed in writing complains to different authorities and ended by believing in a conspiracy between the GP, the technical authorities and the mayor of the municipality.

On the contrary, in the following case of a patient with schizophrenia, we see with the same content of delusion concerning poison condition of tap water, a completely different and idiosyncratic attitude towards it.

Case 4: One of our patients with schizophrenia, who harbors a similar belief about the toxicity of the water did not contact any authorities, but figured out by himself a solution to the problem: he stored water in special containers in “fresh air” before using it for drinking or cooking.

To sum up, schizophrenic delusions are not beliefs about worldly matters, rather they concern a different realm transcending the shared-social world. Therefore, the delusional ‘evidence’ is not concerned with evidence rooted in the shared world and the two attitudes or ways of orientation—even in the case of explicit contradiction—can exist peacefully side by side.

### Auditory verbal hallucinations (AVH)

AVH are one of the characteristic symptoms of schizophrenia. In contemporary mainstream psychiatry, the definition of hallucination is a variant of the classical definition of hallucinations as “perceptions without an object” [30]. However, phenomenological psychiatry has pointed out that AVH in schizophrenia do not possess perceptual features such as a perspectival giveness of the content and temporal contour. To use the phrase of Charbonneau [31], the sensory qualities of hallucinations are merely a “caricature” of the sensorial (see also [32–34]). Hallucinations are given as meaning fragments, often without sensorial quality (“soundless voices,” [3]), the meanings are articulated all at ones without temporal stretch and the patient experiences the voices as an intrusive part of her inner most intimate sphere (hyperproximity). There are several features that point to the fact that hallucinations articulate themselves in another ontological space than that of intentional perceptual life. The patient only rarely confuses her AVH with real acoustic perceptions.

In a recent study of schizophrenia patients suffering from AVH there was a long time interval between the onset of hallucinations and their disclosure to the treating medical personnel [34]. The delay was especially long in patients whose onset of hallucinations was in childhood. The patients typically consider their experiences as “thoughts.” These thoughts often acquire the status of “voices” only at the event of naming (nomination) by the treating clinician. Thus, the hallucinations were primary experienced by the patients in their private immanent sphere where thinking was felt to be at an experiential distance from the sense of subjecthood.

The link to another ontological domain was already emphasized by Schneider who wrote that the significance of psychotic experience in schizophrenia carries for the patient “a sign or message from another world” [35, p. 104].

Case 5: One of our patients wrote to the first author: I have read the text that you have recommended [on phenomenology of thinking]. What surprised me was that our thoughts are separated from each other. That your thoughts belong to you and just to you and my thoughts belong to me and just to me. Since I was a child I have been of the conviction that all peoples’ thoughts and voices were mixed together in a collective whole. From this whole, came my voices, knocking sounds, voices, mumblings, whispers, or this occasional screaming.

It is clear from this report, that the patient’s sense of privacy and mineness of thinking is disturbed. There is an apparent continuity between thinking and hallucinations. Most importantly, the patient ascribes these phenomena to some kind of universal extra-sensorial space. It seems that this mode of experience is habitual for the patient. Some of these aspects are illustrated in the following vignettes.

Case 6: I have always known that this was my place, this was my reality. Away from other people's reality. I live in the shared world just like all humans. And then I also have my own reality. Of course, I know that there is not a man standing there talking to me… It all takes place in my head. I know that. And I am completely aware of that. But to me it is my reality. I have lived like that for years. I really feel that I live in two worlds [34].

Case 7: One of our patients reported the following experience: “…Sometimes I can hear people that I know, talking about me, even if they are not there. It is distressing, because I do not know what to believe. If I have heard my mother talking about me and I confronted her with it when we meet in the evening, she says that she never did that, and then I do not know what I should believe: The things she said to me face-to-face or what I have heard…”.

What is interesting in the patient’s statement is that she does not question the validity of her hallucinatory experience despite the fact that the experience violates all causal laws in our common reality, or differently put, if someone heard their mother's voice, knowing that she was miles away, a pressing question would be to how that was actually possible, given the causal laws in our common reality. The patient does not consider such questions, which for the patient are completely irrelevant, precisely because this experience is given as absolutely undoubtable. Nonetheless, the patient is distressed by this apparent contradiction between her experience and the mother’s statement, which she seems to consider on the equal footing.

In most cases, the hallucinations possess subjectively convincing and a rather immanent character [30]. In other words, the hallucinatory “voices” articulate themselves in the midst of the patient’s most intimate sphere, from which she cannot escape. The patient may feel to be subjected to a complete exposure, where the voices know everything, is omnipresent and yet, at the same time always alien and furtive [36]. Thus, rather than being integrated or “woven into the fabric of the intersubjective world” [14] hallucinations, with Merleau-Ponty’s words “play out on a different stage than that of the perceived world” [37, p. 396].

### Insight into illness

Lack of insight is perhaps the domain of experience where double bookkeeping is most clearly manifest. “Insight into illness” in contemporary psychiatry is defined as an awareness of the illness, its symptoms and consequences [38]. In general terms, lack of insight, is considered as a typical feature of schizophrenia and is responsible for discontinuation of treatment and frequent relapses.

In other words, insight is defined following the medical model and refers to when the patient is aware of suffering from diabetes and its symptoms and long-term risks. We think that this approach to insight is not entirely adequate in the case of schizophrenia [39]. This inadequacy has already emerged in our description of delusional and hallucinatory experiences. Bleuler draws attention to the fact that many so called “cured patients” harbor the original delusional conviction [3]. He mentioned a discharged professor who apparently “corrected” his delusional convictions, but nonetheless dedicated his latest scientific treaties to his delusional mistress. Another similar patient of Bleuler, continued after recovery to add “Lord” after his signature.

Case 8: One of our patients, a woman suffering from remitted schizophrenia and on constant depot anti-psychotic medication participated in a teaching interview. She described in detail her past psychotic episodes of which the first originated with a feeling that her telephone was bugged. She now declared herself to be free of hallucinations or strange ideas, but continued to suffer from lack of energy and social isolation. After the completed interview, the supervisor asked the question, “but misses Hanson why was your telephone bugged in the first place?” and the patient responded, “This question I ask myself too to this very day.” Clearly, the validity of the original delusional experience was entirely intact for the patient.

In our series of patients with auditory hallucinations, none of the patients believed that they suffered from an illness analogous to a somatic disease.

Case 9: “Other people will say that I’m sick but I don’t feel sick. I feel that it is a part of me and that it is just how I am” [34].

Professor Elyn Saks in an article on insight in schizophrenia raises the following puzzling question of how a person really can deny “her illness in the face of flagrant symptoms?” [40]. She gives the following account of her own view on being diagnosed with schizophrenia:

Case 10: “I completely recognized that the things I was saying and doing and feeling would be thought to amount to a diagnosis of schizophrenia; but I thought that it was not true—I did not really have the illness (…) So, my thinking went, I looked like I had schizophrenia (…) but if we knew enough, we would see that I really did not” [40, p. 972].

Apparently, she experiences an access to a deeper layer of reality, not accessible to other people and not accessible to our current scientific methods. Thus, she uses an ontic, mundane terminology to explain her unique access to the ontological dimension.

### Anderssein: “Being different”

One could now ask, whether double bookkeeping is an aspect of a psychotic, e.g., delusional or hallucinatory state. However, many patients in the pre-onset phases of the illness and in schizotypal conditions, experience more subtle alterations of their subjective life and existential attitudes, already in childhood and adolescence, alterations that do not qualify as a flagrant psychotic condition.

Patients with schizophrenia spectrum disorders often experience that their subjective life and relations to the surrounding world are dramatically different from that of their peers’. Despite apparently normal social behavior, they report a sentiment of profound solitude. In other words, the nature of existence may be already altered quite early in life. Patients with schizophrenia spectrum disorders frequently report that they have felt different from others since early childhood or adolescence. In German psychiatry, this feeling is termed “*Anderssein*.” It is a peculiar feeling of being different, in which the feeling of difference precedes finding out what is different [41]. The patients often have difficulties verbalizing the nature of difference and frequently use comprehensive and vague terms such as “I felt wrong”, “I did not fit in”, or “I failed to bond and connect.” It is well described by Japanese psychiatrist Mari Nagai, who presents the following case.

Case 11: I’m somehow in all respects different from others. My facial features, the feeling I express, the environment I was born in … anyway, it is all different. I have to do everything anew from the beginning [42].

Nagai emphasizes that although the patient lists specific features, the feelings of difference is not rooted in any of them but are merely illustrative of an almost ineffable subjective experiences. She contrasts Anderssein with the feeling of difference in what was called neurotic disorders, where the patient is concerned with her difference from “a specific other, unspecified multiple others, or even the others as ‘norms’” [42]. The difference in the neurotic case is anchored in a shared-social world, where the subject finds itself in a particular position in relation to the others. Articulating a difference presupposes a specific (ontic) dimension of comparison, whereas patients with schizophrenia have precisely a difficulty in concretizing the dimension, because the difference does not concern concrete mundane features (ontic), but the very nature of being-in-the-world (ontological dimension). Thus, it is the very sense of being that seems to be different.

In other words, the patient with schizophrenia spectrum disorders experiences his own subjectivity and its existence as profoundly detached from the common intersubjective reality.

The sense of difference may become gradually thematized, for example in childhood, there may be fantasies of being an elf or feelings of being an extra-terrestrial or of not really belonging to one’s family.

Case 12: One of our patients with schizophrenia told us that as a child he felt that it was strange that he was born in this particular place and lived in this particular home with his parents. He doubted that his parents and grandparents were his biological relatives and sometimes he had a feeling that he was not a human being.

In adolescence, this feeling may become associated with feeling to be uniquely chosen, having special abilities, or with preoccupations with metaphysical or philosophical concerns. The patient may have an experience of having a better access to hidden dimensions of reality that are not available to others.

Case 13: One of our patients, a young female diagnosed with a schizophrenia spectrum disorder, said: “I have always felt different […] I have always felt like… that I was chosen to do something spectacular that would change the world. I feel like the Universe has something to do with it in some way. I have experienced a few times to be one with everything, where everything was connected. I was euphoric. I was like ‘Yes! I’m part of the Universe.’ Most of the time I feel like I’m not related or connected to the world.

The sense of access to another ontological realm may persist on a subtle level in the schizotypal patient or may amplify into a moment of revelation with the emergence of flamboyant psychotic symptoms [14]. The experience of penetration into another ontological realm may be followed by cognitive and metaphysical elaboration of this experience with a formation of various delusional explanations, which the French psychiatrist Henry Ey termed “psychotic work” (*travail psychotique*) [18].

## Discussion

The important issue is how to identify and clarify the shared phenomenological aspect of the presented clinical manifestations. We need to emphasize that in these clinical examples we do not merely encounter a psychotic patient, who by necessity finds himself in our common world. It nor isn’t the case of simply harboring conflicting attitudes. As should be clear from the clinical descriptions, psychotic experience cannot simply be understood as a single deficit in the formation of beliefs or the processes of perception. Rather, we encounter something more profound and radically altered, namely a sort of enigma due to the double attitude or double way of relating. More specifically, it seems to us that the patient’s statements somehow express ontological convictions, which appear mysterious for us, but are apparently unproblematic for the patient. It seems that the patients operate with a kind of evidence, which we as observers are cut off from. This aspect, which strike clinicians as something enigmatic is not a symptom in the same discreet manner as thought pressure or flat affect. Rather, it is a phenomenon, which expresses a certain whole implicating a fundamental change in the structure of subjectivity. This fundamental change apparently confers a trait of specificity on the manifold of symptomatic and behavioral manifestations. An experienced clinician can perhaps notice in an atmospheric way that there is something paradoxical and strange in the patient’s expression and her way of being. However, to go beyond this atmospheric stage, we need to conceptualize more clearly, what is at stake in this impression of paradoxicality and enigma.

This phenomenon of enigma and mystery was already a cardinal point in the discussion about madness in the beginning of “modern psychiatry” [43].

[T]he embarrassments, impasses, advances, and discussions in the clinical debates in the nineteenth century and later revolve around this complete elusiveness of madness, which must be thought of at the same time [as afflicting] (…) everything yet without being total or being at the same time always partial and always total [43, p. 33; our translation].

In other words, the paradox consists in a tension between madness understood as a total dissolution of subjectivity, while at the same time finding a preservation of the very same subjectivity. Pinel considered psychosis as a sort of foreign body with an intact subject behind or besides the psychotic symptoms [1]. His successor, Esquirol, had a much more complex view in which the subject was *both divided and unified in a strange fashion* [2]. He emphasized that madness involves a disintegration of the self. According to Swain:

Everything takes place in the interiority of the single and same self, but a self in which the unity is defective, in the manner that he can be at the same time a victim of delusions on the one hand, and be his normal self on the other hand [43, p. 35; our translation].

Esquirol located the essence of psychosis in the very subject, who despite the cleavage or fissure remained experientially unified. Later in the nineteenth century, Morel, another French psychiatrist, used a metaphor of “Dédoublement du sujet” (duplication of subject), which unfortunately later came to be misunderstood. This was misunderstood literally as a kind of two numerically distinct personalities each with a separate subject as in dissociative states. Bleuler’s view of schizophrenia continues to suffer from the same misunderstanding [44].

It is safe to say that some sort of splitting, or rather, disintegration is at stake in subjectivity in schizophrenia. This peculiar paradoxicality of the subject is very clearly described by a Swiss phenomenological psychiatrist, Jakob Wyrsch. He discussed Ida, a patient with schizophrenia:

Ida’s world is larger than the everyday world; her subjective experiences (their content) are objectified as delusions and hallucinations. Contrary to the normal subject, her experience is not superposed in the world in the form of beliefs, assumptions and superstitions; her experiences immediately articulate a status of reality and conviction and occupy a specific space in the world. For an observer, these phenomena are symptoms, but for the patient, it is her own private world, in which nobody can participate [45, p. 23; our translation].

In the case of Ida, we witness two persons, one involved in the everyday world and the other in the delusional world. However, it is not the case of alternation as in the case of multiple personalities. It is only from the observer’s point of view that there are these two personalities; from Ida’s point of subjective experience, these two worlds belong to the same experience. In the acute psychosis, there may be a discontinuity and disintegration of the experience [and perhaps enactment of delusion in the empirical world]. It is not the case in chronic patient like Ida [45, p. 42; our translation; insertion added in square brackets].

This peculiar division in a unified or singular subject has recently been described in an autobiography of a patient with schizophrenia:

Suddenly, often, because of a small trauma such as viewing an episode of the Star Wars in the movies- the mind starts to divide itself to a part, which is dark and evil and which appears as being external to the self, while the part, which one associates with oneself seems to be blocked in a corner of the consciousness and submits to the power of the strange oneself, who is the self without being it entirely.

It is here that there is the essence of schizophrenia. It is not a duplication of personality where the interior persons succeed each other without mutual awareness; it is rather a division of the thinking, where the two parts brush each other and collide with each other [46, p. 74; our translation].

To understand this peculiar fissure of subjectivity, we have to address the nature of the patient’s experiential evidence. Esquirol claimed that the patient’s conviction is stronger than his reflective judgment: “You are right, said to me a patient, but you cannot convince me” [47, p. 42]. Obviously, as we indicated above in the discussion of primary delusional conviction the evidence is of an affective and not of a cognitive nature. A grasp of the phenomenological structure of this evidence appears to be the key to understanding of the original articulation of the psychotic experience and double bookkeeping.

It seems that the patient’s evidence does not come from an intentional experience, like seeing an object, listening to an argument or thinking about a potential solution to a problem, which may be all fallacious or questioned. It is an experience that is completely apodictic, leaving no room for doubt and therefore carrying with it a complete conviction and incorrigibility. This kind of experience is revelatory, in other words an immanent experience arising in the midst of the patient’s innermost subjectivity or self [14]. In this revelation, the what of the experience (content) seem to be identical to the how (mode or form). Differently put, the experience does not have an ‘object’ as in the ordinary intentional subject/object structure of experience, or does not possess the noetic-noematic structure. The French phenomenologist Michel Henry talks here about auto-affection [48]. For Henry auto-affection is an internal affective pulse of subjectivity that is entirely immanently generated and non-intentional. Henry considers that phenomenality (appearing: articulation of conscious experience) of auto-affection or affectivity has a foundational ontological status, whereas the phenomenality of intentionality possesses a secondary and derivative role.

Henry sees in the auto-affection of affectivity the essence of self-manifestation, self-awareness or *ipseity* (in Latin “ipse” means self or itself). All intentional acts (e.g., perceiving, imagining, remembering, etc.) are self-aware or self-conscious because of being embedded and founded upon self-affection. Phrased differently, all intentional conscious acts articulate themselves in first person perspective as my experiences because they are permeated by the auto-affective dimension of immanence. Thus, whereas all intentional appearance (e.g., a concept or percept) may be false or illusory, the immanent, auto-affective “lived” (*erlebt*; *vecu*) dimension of appearance can never be so. Henry ascribes this original insight to Descartes:

I am now seeing light, hearing a noise, feeling heat. But I am asleep, so all this is false. Yet, it *certainly seems to me* that I see, hear and am warmed. This cannot be false [49; our italics].

It is clear that Descartes’ perceptual experiences must have been false because he was asleep. However, that it *seems* to him to have perceived cannot be false and cannot be doubted. This “seeming” is according to Henry an example of auto-affection, that is, the subjectivity’s intrinsic affectivity [48, 50]. If in my dream I am overwhelmed by a feeling of profound sadness, I cannot say that this feeling was fake or a kind of illusory representation. This feeling possesses an unquestionable first personal ontological reality [51]. It is the auto-affective articulation of self and self-experience, most clearly expressed in intransitive affective states. In this way, we can understand the schizophrenic primary and generative psychotic experience as being originally an alteration or breach of auto-affection.

Henry is insisting on the non-relational nature of self-affection, precluding any division or chasm, though he also emphasizes the dynamism and vital rhythm of subjectivity [52]. We have elsewhere discussed the profound self-alienating experiences occurring in schizophrenia (viz. self-alterization) as conditioned by a potential alterity implicit in the dynamic structure of subjectivity [41]. In other words, self-affection entails a ceaseless differentiation and merging of affective moments [53]. These affective moments have the potential to form an alterity in this process of bifurcations and fusions. This pre-reflective circular movement thus offers a chasm or fissure which normally becomes rapidly sealed ensuring the sense of self-coincidence. In schizophrenia spectrum disorders, this fissure remains unintegrated, allowing for the emergence of the characteristic self-alterization. The latter implies that the chasm of auto-affection thus allows moments of subjectivity to manifest a sense of affective otherness and independence. This is the essence of the originary psychotic experience with a revelatory character. It involves a feeling of a breakthrough to another ontological dimension that is experience with varying intensity and conceptual elaboration, ranging from a sense of having a unique immanent life to a solipsistic omnipotence, merging with the divine, or to other, transcendent realities. The sense of otherness within the immanence of the self becomes eventually intentionally structured with delusional or hallucinatory content. Ey describes it in the following way:

The experience of dis-structuration of the field of consciousness entails a fundamental experiential modification of the subjective–objective relation… This is the pathic [affective] (sensible) coefficient of this relation which is affected in the psychotic experience; we call this phenomenon in its generality the experience of alterity; the modality of feeling what is me or mine is changed. It consists of feeling oneself an Other… it consists to gradually experience what belongs to the subject, as becoming increasingly alien and ultimately other within the subject himself [18, *Tome I*, p. 417; our translation].

We have elsewhere suggested [54–57] that the fundamental trait disorder in schizophrenia consists in an instability of first-person perspective, i.e., a disorder of basic self. The notion of basic self implies that all our experiences are self-saturated (i.e., self-affecting), articulating themselves in first person perspective [58]. Experiences are self-affecting, assuring a sense of self-coincidence and affective self-presence [14, 59]. A correlated aspect of the disorder of basic self is an unstable tacit or pre-reflective relation to the social, environing world, i.e., “common sense.” It results in an instability of the immediate grasp of contextual meanings and an unquestionable realness of the perceptual world [60–62]. In other words, the articulation of selfhood and the basic intersubjective attunement are co-dependent phenomena. As Merleau-Ponty stated it, subjects and objects are “two abstract moments of a unique structure, namely presence” [37, p. 494].

In sum, we claim that the disorder of basic self (instability of first-person perspective) leads to chasmic disruptions of the auto-affective homogeneity of the self. The immanent fissure allows for the emergence of an otherness within the very immanence of the self (the process of alterization). This immanent otherness is the kernel of the psychotic experience. This kernel is felt as a break-through to another ontological dimension and evolves into various psychotic phenomena accompanied by double bookkeeping. From the observer’s point of view, double bookkeeping imbues the clinical picture with atmospheric paradoxicality, which reflects the underlying core of structural disorder of subjectivity.

## Implications

We will point to four interrelated implications of the concept of double bookkeeping and its phenomenological structure: (1) diagnostic (2) epistemological (3) therapeutic and (4) pathogenetic research.

A phenomenological grasp of double bookkeeping may help the clinician to comprehend the paradoxical strangeness of the patient in more clear and concrete clinical terms, perhaps concretizing his original atmospheric impression. The clinician should be especially alerted to a possible double bookkeeping when the patient’s statements and behaviors manifest certain inconsistences, discordances or apparently paradoxical reasonings, expressions and attitudes. The phenomenon of double bookkeeping appears to be quite characteristic for the schizophrenia spectrum disorders as illustrated above.^3^ However, it must also be clear that a phenomenological grasp of double bookkeeping does not allow for a creation of an “operational” diagnostic rule: the phenomenon is too complex to be converted into a simple symptom or sign that could be elicited by a structured or preformed interview-questions. It is characteristic of the phenomenon of double bookkeeping that it expresses multiple meaning-aspects of a fundamentally altered existential position. Therefore, it demands a comprehensive exploration of the patient’s experiential life.

On the epistemological level, the formation of psychotic reality and its apodictic character for the patient point to the fact that the descriptive notion of psychosis as a defective reality testing or harbouring false epistemic assumptions cannot be maintained [65]. The alternative ontological framework originates in an undeniable first personal experience in the patient’s most intimate sphere and can therefore not be merely viewed as a false representation of reality. This complication of the view of psychosis as an epistemic error has been, as mentioned, repeatedly emphasized in phenomenological literature (e.g., Jaspers, Tatossian, Spitzer and Parnas). Thus, the phenomenon of double bookkeeping seems to emphasize the fact that psychopathological manifestations of schizophrenia spectrum cannot be adequatly addressed by the medical notions of symptoms and signs [30].

This leads us to the therapeutic aspect. In dealing with non-acute psychotic patients, it is precisely paramount not to dismiss their experiences as being merely errors or fictions. Rather, it is important to acknowledge their first personal reality and existential significance and help the patients to negotiate a balance between the two incommensurable attitudes (see also [66, 67]). This may indicate that to help the patient to find her own way entails negotiating the balance between social contacts and solitary interests and activities. Furthermore, most clinicians are familiar with the fact that patients often resist medication because it flattens out their immanent life. Here, the treating psychiatrist needs to realize that this immanent life has an existential value for the patient and for this reason, she has to adjust the medication in cooperation with the patient. These considerations are implicitly reflected in a very important statement by Jaspers:

[The patient’s] world has changed to the extent that a changed knowledge of reality so rules and pervades it that any correction would mean a collapse of being itself, in so far as it is for him his actual awareness of existence. Man cannot believe something that negates his existence [17, p. 105].

Finally, in terms of pathogenesis, it seems to us that empirical research should increasingly focus on the basic, generative aspect of schizophrenia such as disorders of selfhood and intersubjectivity instead of studying pathogenetically distant phenotypic features such as flamboyant psychotic phenomena or negative symptoms. There is a consistent evidence that schizophrenia is a developmental disorder, but the research conducted so far has neglected the psychological vicissitude of selfhood and sociality [68, 69]. Such a new approach should take into account developmental aspects of subjectivity and sociality not only in neurobiological, but also psychological terms [70].

A limitation to our exposition is the lack of discussion of human rationality and its variations. Additionally, we have focused on the issue of selfhood, but we did not explore in depth the role of basic intentionality and intersubjectivity.

## Data Availability

Not applicable.

## Footnotes

1 The distinction between these two levels of reality has an obvious affinity with Husserl’s distinction between the natural attitude (our everyday attitude towards the world) and the transcendental attitude as well as to Heidegger’s distinction between the ontic level (the level of mundane existing Beings) and the ontological level (the level of Being as such). We will not pursue these affinities in an explicit way in our text.

2 The notion of belief in itself is quite vague, but in our common sense and psychiatric use of the term, it is ascribed to the statements of knowledge and conviction: “I believe such and such”. Several anthropologists have pointed to the fact to a quite polysemic nature of the notion of belief [27, 28].

3 The dutch psychiatrists Rümke talked about “praecox feeling,” which he however was unable to specify more closely [63, 64]. We believe that the recognition of double bookkeeping contributes to this atmospheric “praecox feeling.”.
